# Exploring Angiotensin II and Oxidative Stress in Radiation-Induced Cataract Formation: Potential for Therapeutic Intervention

**DOI:** 10.3390/antiox13101207

**Published:** 2024-10-08

**Authors:** Vidya P. Kumar, Yali Kong, Riana Dolland, Sandra R. Brown, Kan Wang, Damian Dolland, David Mu, Milton L. Brown

**Affiliations:** 1 Armed Forces Radiobiology Research Institute, The Uniformed Services University of the Health Sciences, Bethesda, MD 20889, USA; vidya.kumar.ctr@usuhs.edu; 2 Department of Biomedical and Translational Sciences, Macon & Joan Brock Virginia Health Sciences at Old Dominion University, Norfolk, VA 23507, USA; kongy@odu.edu (Y.K.); wangk@odu.edu (K.W.); mud@odu.edu (D.M.); 3 Trocar Pharma Inc., 8101 Sandy Spring Rd., Suite 300-W9, Laurel, MD 20707, USA; riana.dolland@trocarpharma.com (R.D.); damian.dolland@trocarpharma.com (D.D.); 4 LensCrafters, Inc., 110 Mall Circle, Suite 2001, Waldorf, MD 20603, USA; brownfamily61@verizon.net; 5 Leroy T. Canoles, Jr. Cancer Research Center, Macon & Joan Brock Virginia Health Sciences at Old Dominion University, Norfolk, VA 23507, USA; 6 Department of Internal Medicine, Macon & Joan Brock Virginia Health Sciences at Old Dominion University, Norfolk, VA 23507, USA

**Keywords:** angiotensin II, oxidative stress, radiation-induced cataracts, lens transparency, therapeutic intervention

## Abstract

Radiation-induced cataracts (RICs) represent a significant public health challenge, particularly impacting individuals exposed to ionizing radiation (IR) through medical treatments, occupational settings, and environmental factors. Effective therapeutic strategies require a deep understanding of the mechanisms underlying RIC formation (RICF). This study investigates the roles of angiotensin II (Ang II) and oxidative stress in RIC development, with a focus on their combined effects on lens transparency and cellular function. Key mechanisms include the generation of reactive oxygen species (ROS) and oxidative damage to lens proteins and lipids, as well as the impact of Ang II on inflammatory responses and cellular apoptosis. While the generation of ROS from water radiolysis is well established, the impact of Ang II on RICs is less understood. Ang II intensifies oxidative stress by activating type 1 receptors (AT1Rs) on lens epithelial cells, resulting in increased ROS production and inflammatory responses. This oxidative damage leads to protein aggregation, lipid peroxidation, and apoptosis, ultimately compromising lens transparency and contributing to cataract formation. Recent studies highlight Ang II’s dual role in promoting both oxidative stress and inflammation, which accelerates cataract development. RICs pose a substantial public health concern due to their widespread prevalence and impact on quality of life. Targeting Ang II signaling and oxidative stress simultaneously could represent a promising therapeutic approach. Continued research is necessary to validate these strategies and explore their efficacy in preventing or reversing RIC development.

## 1. Introduction

Radiation-induced cataracts are increasingly recognized as a significant public health issue due to their substantial impact on vision and overall quality of life. Cataracts, which result in the clouding of the eye’s lens and lead to impaired vision, are a major global cause of vision impairment. In the United States alone, over 20.5 million individuals aged 40 and older are affected by cataracts, with more than 6.1 million undergoing cataract surgery each year [1]. The prevalence of cataracts escalates with age, affecting more than 50% of people by age 80, either through the presence of cataracts or prior surgical intervention [2]. A significant proportion of these cases are linked to exposure to radiation exposure because the lens of the eye is one of the most radiosensitive tissues [3]. There are five types of common radiation exposures: (i) ultraviolet radiation [4], (ii) cancer radiotherapy, and (iii) radiation exposure in occupational settings, (iv) natural disasters, and (v) military or terrorist activity [5]. Extensive research has established a clear association between radiation exposure and cataractogenesis [6,7,8]. While IR is a well-known cause of cataracts, the exact pathogenesis of radiation-induced cataracts remains incompletely understood. Individuals exposed to radiation prenatally or during childhood are particularly vulnerable, with a median time of 27.6 months post-radiation treatment required for cataracts to develop [9,10]. Therefore, effective radiation protection for the eye lens is crucial in fields where daily exposure to radiation is a concern, such as in medicine, aerospace, and air travel [11,12,13,14,15,16,17,18].

Cataracts significantly impair vision by obstructing and scattering light as it reaches the retina [19], resulting in symptoms such as blurred vision, glare, and decreased contrast sensitivity [20]. Cataracts are anatomically classified into three subtypes: nuclear, cortical, and posterior subcapsular [21]. Among the various types of lens cataracts induced by ionizing radiation, posterior subcapsular cataracts (PSCs) are the most prevalent [22]. Despite some studies on the epidemiology and biology of radiation-induced PSCs, the mechanisms underlying their formation and their dose dependency remain uncertain [23]. Nuclear and cortical cataracts arise from pathological changes within the lens fiber cells, while posterior subcapsular cataracts are linked to abnormalities in the lens’s germinative zone [24]. Cataracts can also manifest as late effects following survival from acute radiation syndrome (ARS) [25]. Accumulating evidence indicates that ionizing radiation (IR)-induced cataract formation may be linked to oxidative stress [26,27], with studies showing that diets supplemented with antioxidants can provide preventative effects in laboratory animals [28]. Additionally, total body irradiation has been associated with a chronic decrease in antioxidant levels, further contributing to the risk of cataract development [29]. Understanding these connections is essential for developing strategies to mitigate radiation-related ocular complications. Consequently, oxidative stress/ROS is a major area we focus on in this report.

IR is a well-established risk factor for cataract formation, with radiation-induced cataractogenesis involving complex mechanisms such as cellular damage and oxidative stress [30]. Radiation-induced cataracts are typically localized to the posterior region of the lens [31,32], with cortical cataracts being the next most frequently observed type [33]. This posterior predominance is due to the posterior subcapsular region’s heightened vulnerability to radiation [14,34], leading to oxidative stress, DNA damage, and cellular apoptosis. Mathis et al. found that radiation-induced cataracts frequently manifest as posterior subcapsular opacities, underscoring the specific susceptibility of the posterior lens region to IR [35]. Similarly, others support this regional vulnerability and identified posterior subcapsular opacities as the most common type of cataract following radiation therapy [30,36].

In studies involving rabbits and rodents exposed to high doses of irradiation (50–60 Gy) focused on the central part of the lens while shielding the rest of the body, slit lamp evaluations using the Merriam-and-Focht scoring system showed a significant decrease in mitotic activity in the anterior lens epithelium’s central region, or germinative zone, which lasted up to 6 months. Despite this, cataract development was observed, starting with the formation of central posterior subcapsular vacuoles and dots (stage 1+). This was followed by an increase in posterior cortical opacity and the emergence of central anterior opacity (stage 2+). By stage 3+, changes were observed in both the anterior and posterior lens cortex, ultimately leading to complete lens opacity (stage 4+). Initial formation of posterior subcapsular vacuoles and dots is believed to be due to epithelial cells that fail to differentiate into lens fiber cells and instead migrate towards the posterior lens capsule [37].

RICF occurs when IR damages and destroys the lens epithelium, creating gaps that allow reactive oxygen species (ROS) to infiltrate the lens and cause oxidative damage to crystallins. This damage leads to protein denaturation, aggregation, and the formation of multilamellar bodies, which are primarily responsible for lens opacification [38] and accelerated cataract progression [39].

Ang II, a peptide primarily known for its role in regulating blood pressure, is known to be involved in oxidative stress and inflammatory responses [40,41,42]. IR elevates Ang II levels in certain tissues [43], and elevation of the ocular renin–angiotensin system (RAS) is known to exacerbate lenticular oxidative stress, which may lead to cataract formation [44]. The interaction between elevated Ang II and ROS creates a critical pathway in the pathology of RICF, underscoring the need for dually targeted therapeutic interventions. Understanding these mechanisms provides a valuable opportunity to develop therapies aimed at mitigating the effects of radiation exposure and preventing cataract onset. By addressing both roles of Ang II and ROS, novel strategies could be formulated to preserve lens integrity and enhance outcomes for individuals at risk of cataract formation from radiation exposure.

The objectives of this article are twofold: first, to explore the roles of Ang II and oxidative stress in the development of radiation-induced cataracts, and second, to identify potential therapeutic strategies to prevent or mitigate cataract formation. We aim to discuss the contribution of Ang II to oxidative stress and lens damage in the context of radiation exposure as a separate mechanism from ROS created by radiolysis of water, thereby providing a comprehensive understanding of the molecular mechanisms involved in cataractogenesis. An expected outcome of this study is to excite interest in creating molecular countermeasures that target both Ang II signaling and oxidative stress to mitigate RICF, ultimately improving ocular health for individuals exposed to IR.

## 2. Renin–Angiotensin System (RAS)

**Figure 1:**
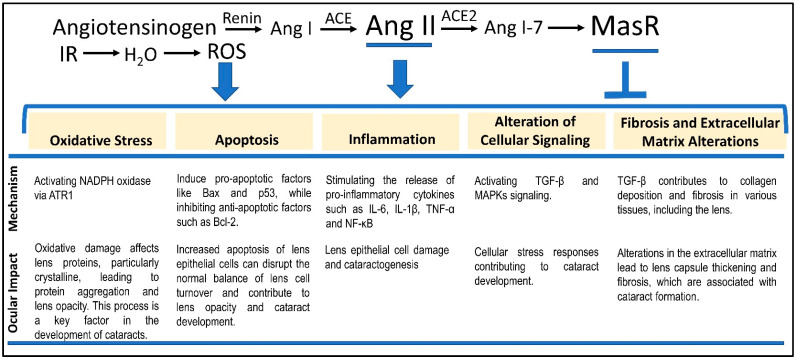
Five major mechanisms mediating Ang II-dependent RICF. We discuss the five RICF mechanisms connected to both the RAS signaling pathway and the IR-induced ROS. Each of the five mechanisms is derived from the interplay between radiation-dependent enhancement of Ang II signaling and ROS. While AT1R mediates the activities of Ang II, MasR counters the Ang II-induced phenotypes via interactions with Ang 1–7. We suggest that a combination of MasR agonism and ROS suppression may be considered a fruitful direction for RIC countermeasure development.

The RAS plays a pivotal role in regulating blood pressure and fluid balance, with Ang II serving as a central mediator in this system [45]. The RAS is initiated by the release of renin from the kidneys in response to low blood pressure, reduced renal perfusion, or low sodium levels. Renin catalyzes the conversion of angiotensinogen, a protein produced by the liver, into angiotensin I (Ang I) (Figure 1). Subsequently, Ang I is converted into Ang II by angiotensin-converting enzyme (ACE), primarily located in the lungs and other tissues, marking a crucial step in the RAS.

Ang II is a crucial peptide hormone involved in various physiological processes, primarily related to cardiovascular and renal function. It is a key regulator of blood pressure and fluid balance through several mechanisms including vasoconstriction, fluid retention, sympathetic nervous system activation, and cellular growth and remodeling [46]. The physiological effects of Ang II are mediated through several mechanisms. Ang II acts as a potent vasoconstrictor by binding to AT1Rs on the smooth muscle cells of blood vessels, leading to vessel constriction, increased systemic vascular resistance, and elevated blood pressure [47]. Additionally, Ang II stimulates the release of aldosterone from the adrenal cortex, which enhances sodium and water reabsorption in the kidneys. This process increases blood volume and raises blood pressure [48]. Furthermore, Ang II influences cellular growth and tissue remodeling through signaling pathways such as NF-κB and MAPK. It promotes inflammation, fibrosis, and cellular apoptosis, which are associated with pathological changes in various tissues [49].

Ang II exerts its effects by binding to AT1 and AT2 receptors throughout the body, leading to vasoconstriction, stimulation of aldosterone secretion, and modulation of sympathetic activity. These actions collectively work to restore blood pressure and fluid balance [50]. The RAS is regulated through negative feedback mechanisms, where elevated levels of Ang II inhibit renin secretion, thereby limiting further production of Ang II [51].

## 3. Ang II and RICF

Ang II extends its influence beyond cardiovascular regulation to impact ocular pathologies, including radiation-induced cataracts. IR generates ROS by radiolysis of water, which can lead to significant oxidative stress and damage to lens proteins. Ang II exacerbates this oxidative damage through multiple mechanisms, including enhanced ROS production, inflammation, apoptosis, and lens fibrosis.

The role of Ang II in cataracts have been studied in rodents [44]. Ang II increases ROS levels by activating NADPH oxidase, which contributes to oxidative stress and subsequent damage to lens epithelial cells and crystalline proteins, thereby promoting cataract formation [52]. Additionally, Ang II induces inflammatory responses by activating the NF-κB signaling pathway and increasing the release of pro-inflammatory cytokines [53], which could further aggravate cataract development. It also could trigger apoptosis in lens epithelial cells, compounding the damage and accelerating cataract progression [54]. Furthermore, Ang II promotes lens fibrosis through the activation of TGF-β signaling, which alters the extracellular matrix composition and leads to lens capsule thickening and opacification [55].

Understanding the role of Ang II in radiation-induced cataracts underscores the potential for targeted therapeutic interventions. Strategies that modulate Ang II activity or its downstream effects could provide promising approaches to prevent or treat cataracts in individuals exposed to radiation.

## 4. Ang 1–7/Mas Receptor Interplay

The Mas receptor (MasR), a G protein-coupled receptor, has been extensively studied for its functions within the renin–angiotensin system (RAS). Activated primarily by angiotensin (1–7) (Ang 1–7), a peptide derived from angiotensin II through the action of ACE2, MasR is known to mitigate many of the harmful effects associated with Ang II [56], maintaining cardiovascular homeostasis (Figure 1). Localization of Mas receptors in the eye suggests that it may have an important role in physiological processes [57]. However, the specific role of MasR in the pathogenesis of RICs remains largely unexplored, especially concerning how alterations in Ang 1–7 levels may influence cataract development. Additionally, a decline in the ACE2/Ang 1–7 pathway’s activity could critically impact ocular tissues and other radiation-damaged areas. A corollary is that the balance between the activation of AT1R and MasR is essential in modulating the overall impact of Ang II on various tissues, including the eye [58].

Ang (1–7) functions to counterbalance Ang II, likely by downregulating the phosphorylation of MAPK pathways (p38, ERK1/2, and JNK), indicating an upstream protective role in RICs by modulating these signaling cascades. In contrast to Ang II, which predominantly causes vasoconstriction and pro-inflammatory effects via AT1R and AT2R, MasR promotes vasodilation and offers protective cardiovascular effects through pathways that enhance nitric oxide production and reduce oxidative stress.

In the context of RICs, the Mas receptor is pivotal. Radiation exposure disrupts the balance between Ang II and Ang 1–7, leading to diminished activation of the Mas receptor. Emerging insights into RAS biology underscore MasR’s potential to counteract the vasoconstrictive and inflammatory effects of Ang II. Increased MasR activity could mitigate the adverse effects of elevated Ang II levels, positioning it as a promising therapeutic target for managing RICs, as well as fibrotic, oxidative dysfunction, and hypertensive disorders. Ultimately, the Mas receptor’s influence extends beyond RICs, with its capacity to promote vasodilation and counteract fibrosis, suggesting its broader therapeutic potential.

## 5. Radiation-Stimulated Ang II Expression and Radiation-Induced Cataracts

The lowest cumulative IR dose to the lens of the eye that can produce a progressive cataract is approximately 2 Gy [59]. Mechanistically, IR exposure has been shown to stimulate increased expression of Ang II [43], exacerbating tissue damage and contributing to radiation-induced tissue injury. The interplay between radiation, Ang II, and cataractogenesis involves these five critical mechanisms—(i) oxidative stress, (ii) inflammation, (iii) apoptosis/protein aggregation, (iv) alteration of cellular signaling, and (v) fibrosis. Below, we discuss how Ang II contributes to RICF through each of the five mechanisms (Figure 1).

### 5.1. Oxidative Stress

The oxidative stress caused by Ang II affects lens epithelial cells by inducing damage to other cellular macromolecules, including lipids, proteins, and DNA. This damage compromises the integrity and function of the lens, contributing to cataract formation [60]. In animal models, inhibition of Ang II signaling has been shown to reduce oxidative damage and preserve lens transparency, further supporting the link between Ang II, oxidative stress [61], and cataractogenesis [44].

Alternatively, radiation may directly produce ROS via radiolysis of water, leading to oxidative stress in lens cells. A further increase in Ang II by radiation would exacerbate this oxidative damage by promoting ROS generation through pathways such as NADPH oxidase activation [62]. This enhanced oxidative stress damages the lens, which in turn leads to damage of cellular components, including DNA and proteins, contributing to cataract formation. Studies have shown that elevated levels of ROS result in the oxidation and aggregation of lens proteins such as crystalline, which are central to lens opacity [63].

**Figure 2:**
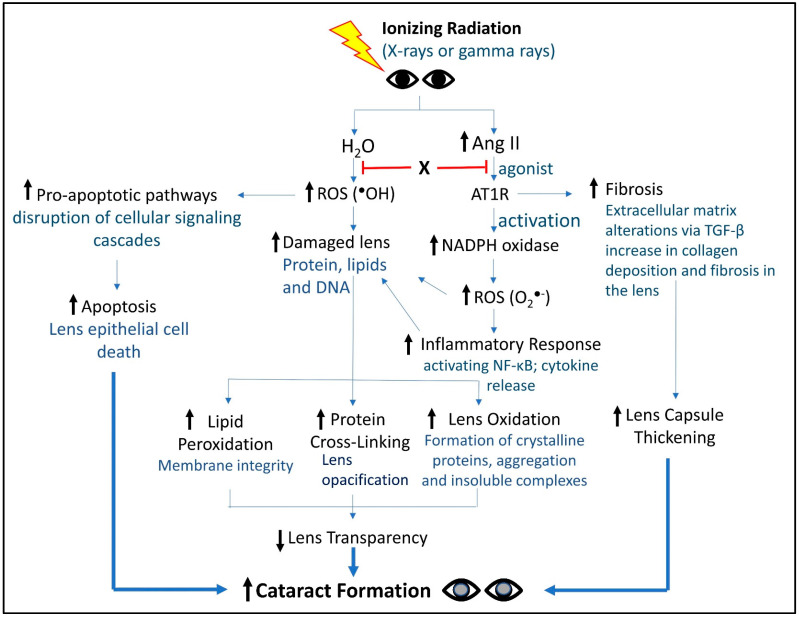
Holistic view of key molecules and signaling events from IR to cataract formation. This schematic captures the activities and events connecting IR to RICF. X represents a small molecule countermeasure we are developing against RICF. As a bi-functional, single small molecular entity, X was designed to tame both Ang II signaling and ROS production, two major activities contributing to RICF. In preliminary animal-based studies, X demonstrated promising activities in suppressing RICF [65]. Future work will be required to further develop X as a bona fide RICF countermeasure.

The multifaceted roles of Ang II in lens pathology have been recently examined [60], with a particular focus on oxidative stress. The report highlights how Ang II contributes to cataract formation through both direct and indirect mechanisms. Directly, Ang II enhances oxidative stress by increasing the production of ROS in lens epithelial cells, which leads to damage and aggregation of lens proteins, ultimately impairing lens transparency. Indirectly, Ang II promotes inflammatory responses and activates apoptotic pathways, further exacerbating cellular damage and cataract development. Evidence in other tissues suggests that Ang II’s dual roles—both as a mediator of oxidative stress and an activator of inflammatory processes [64]—create a synergistic effect that accelerates tissue pathology, thus underscoring the importance of preventing or slowing cataract progression by mitigating oxidative stress and inflammation. Altogether, Ang II exacerbates oxidative damage by enhancing ROS production and inflammatory responses, which synergistically affect lens transparency and promote cell apoptosis. These mechanisms collectively contribute to cataract development, underscoring Ang II as a significant target for potential therapeutic interventions. A summary of the dual role of Ang II and ROS in cataractogenesis is illustrated in Figure 2.

### 5.2. Apoptosis and Protein Aggregation

The pathological role of Ang II in lens epithelial cells, lens transparency, and fibrosis is becoming increasingly recognized. Ang II, through activation of AT1R, exacerbates cell apoptosis in lens epithelial cells [58] via upregulation of pro-apoptotic proteins like Bax and downregulation of anti-apoptotic proteins such as Bcl-2 [66] (Figure 1). Ang II-induced oxidative stress activates inflammatory pathways that further exacerbate cell apoptosis. Specifically, Ang II activation leads to the stimulation of NF-κB (nuclear factor kappa-light-chain-enhancer of activated B cells) [67], which leads to the release of pro-inflammatory cytokines, thereby amplifying cellular damage and apoptosis.

The role of Ang II in the mechanism of diabetic cataracts, which may parallel RICF, suggests that an increase in angiotensin II in the lens promotes oxidative stress-related factor production [68]. Ang II also affects protein aggregation in the lens, a key factor in cataract formation. The oxidative stress induced by Ang II may lead to modification and aggregation of lens proteins, particularly crystalline. Protein oxidation alters the structural integrity of crystalline, resulting in its aggregation and the formation of insoluble complexes that contribute to lens opacification [38]. Ang II-induced oxidative stress has been shown to contribute to protein aggregation [69], a key factor in impaired lens transparency and cataract progression [70,71]. Thus, Ang II’s involvement in cellular apoptosis and protein aggregation may be crucial in driving cell death and contributing to the loss of lens epithelial cells, which is a key factor in cataract formation.

### 5.3. Inflammation

In the eye, radiation exposure triggers inflammatory responses that are further intensified by Ang II. Ang II enhances these inflammatory responses by binding to the AT1R, which amplifies oxidative stress and inflammation through the activation of NADPH oxidases [72] (Figure 2). Additionally, Ang II activation leads to increased release of inflammatory cytokines and stimulation of NF-κB [67] and AP-1 (activator protein 1), which initiate the transcription of various pro-inflammatory genes [73]. This heightened activation results in the release of pro-inflammatory molecules, causing cellular damage, apoptosis [74], and inflammation [75], driving cataract development [76].

### 5.4. Alteration of Cellular Signaling

The mechanisms by which Ang II contributes to cataract formation are dependent on receptor activation. A major consequence of receptor activation is the cellular signaling events triggered by activated receptors. Ang II primarily acts through AT1Rs, which are coupled with G-proteins that activate various downstream signaling pathways. AT1R signaling directly activates key signaling pathways for cell growth and hypertrophy including JAK/STAT (janus kinase/signal transducer and activator of transcription) [77,78], ERK (extracellular-signal-regulated kinase) 1/2 [79], MAPK (mitogen-activated protein kinase) [80], and EGFR (epidermal growth factor receptors). Ang II induces fibronectin synthesis and TGFβ (transforming growth factor beta) activity to promote fibrosis and extracellular matrix (ECM) formation [81] (Figure 1). These signaling pathways are crucial for the cellular responses to Ang II and contribute to the pathological processes leading to cataract formation.

### 5.5. Fibrosis

TGF-β signaling plays a well-established role in fibrosis which describes the formation of excessive fibrous connective tissues [82]. At the cellular level, irradiation prompts an increase in TGF-β expression (Figure 1). This can lead to fibrosis [83], which may be characterized by excessive collagen production, tissue hardening or scarring, and impaired function. Ang II stimulates fibrogenesis on cultured human tenon’s capsule fibroblasts [84] and could play a role in lens capsule thickening and fibrotic changes in RICF. Chronic exposure to Ang II could also affect the differentiation and function of lens fiber cells, leading to changes in the lens’s optical properties. The altered protein dynamics in lens fiber cells further exacerbate cataract formation by promoting the aggregation of lens proteins [85]. Understanding the mechanisms by which radiation-induced Ang II release contributes to cataractogenesis highlights the Ang II signaling pathway as a potential therapeutic target. Modulating Ang II activity or its downstream effects could offer strategies to mitigate radiation-induced cataracts and improve patient outcomes.

## 6. Summary of Importance of ROS and Ang II in RICF

We provide four major concepts regarding how ROS and Ang II contribute to RICF:

(1) ROS are key players in cellular damage and cataractogenesis.
(2) IR leads to an increase in ROS, resulting in oxidative stress that damages lens proteins and lipids. This oxidative damage is a primary contributor to the aggregation of lens proteins, such as crystalline, which is essential for maintaining lens transparency. Elevated ROS levels are also implicated in cellular apoptosis, further compromising lens integrity and accelerating cataract progression.
(3) IR also leads to an increase in Ang II, which exacerbates oxidative stress by activating AT1Rs on lens epithelial cells and enhances ROS production. A net effect is a feedback loop that perpetuates oxidative stress and inflammation, ultimately triggering inflammatory pathways that lead to further lens cellular damage and promote RICF.
(4) The interplay between Ang II and ROS is critical for RICF, exposing an entry point for therapeutic intervention. Indeed, we have designed a single small molecular entity (X, Figure 2) to target both Ang II signaling and ROS production. In preliminary animal-based studies, X demonstrated promising activities in suppressing RICF [65]. Continued research is necessary to validate the efficacy of the two-pronged strategies to counter RICF.

## 7. Discussion

RICs present a significant public health concern, especially as our understanding of the mechanisms underlying their formation deepens. The interplay between radiation exposure, oxidative stress, and the RAS, particularly the role of Ang II, highlights critical pathways for therapeutic intervention. (I) The Role of Oxidative Stress—Oxidative stress, primarily mediated by ROS, is a pivotal factor in cataractogenesis. IR generates ROS through the radiolysis of water, which initiates a cascade of cellular damage, particularly to lens epithelial cells. This damage leads to protein modifications, denaturation, and aggregation, especially of crystallins, which are crucial for maintaining lens transparency. The evidence suggests that the exacerbation of oxidative stress by Ang II not only promotes cataract formation but also enhances the severity of the condition. Inhibition of Ang II signaling has shown potential in reducing oxidative damage and preserving lens integrity, which indicates that targeting oxidative stress through Ang II modulation could provide a viable strategy for mitigating RICs. (II) Ang II: A Dual Role—Ang II is not merely a regulator of cardiovascular physiology; its involvement in ocular pathologies, particularly RICs, underscores its broader implications in health. The elevation of Ang II levels following radiation exposure exacerbates oxidative stress and induces inflammatory pathways that contribute to lens damage. Ang II’s activation of AT1 receptors leads to the upregulation of pro-apoptotic factors and inflammatory cytokines, compounding the risk of cataract formation. This dual role of Ang II—as both a promoter of oxidative stress and a mediator of inflammatory responses—highlights the necessity for a comprehensive approach in therapeutic interventions. (III) Mechanistic Insights into Cataractogenesis—The mechanisms by which Ang II contributes to RICs are multifaceted. The interplay of oxidative stress, apoptosis, inflammation, and fibrosis provides a robust framework for understanding how radiation exposure exacerbates lens pathology. Notably, the activation of signaling pathways such as NF-κB and TGF-β by Ang II further supports a pro-fibrotic environment that leads to lens capsule thickening, a precursor to cataract development. The observations regarding the prevalence of posterior subcapsular cataracts in radiation-exposed individuals align with these mechanistic insights, emphasizing the need for targeted therapies aimed at disrupting these pathways. (IV) Therapeutic Implications—Given the critical involvement of both ROS and Ang II in cataract formation, therapeutic strategies that target these pathways could be highly beneficial. The development of small molecular entities, such as the discussed compound X, which can simultaneously inhibit Ang II signaling and reduce ROS production, represents a promising direction for future research. Such a dual-target approach could mitigate the effects of radiation exposure and slow the progression of cataracts. Preliminary findings suggest that this strategy may yield significant protective effects, warranting further investigation.

## 8. Synthesis, Limitations, and Impact

This research elucidates the significant role of Ang II and oxidative stress in the development of RICs, underscoring their implications for ocular health in populations exposed to ionizing radiation. The findings demonstrate that Ang II not only mediates blood pressure regulation but also exacerbates oxidative damage and inflammation within the lens, leading to cataractogenesis. Notably, the unique localization of radiation-induced cataracts, primarily affecting the posterior region of the lens, is highlighted, emphasizing the need for targeted monitoring and preventive measures in at-risk populations, including cancer patients and occupationally exposed individuals.

A critical gap identified in the current body of knowledge is the limited understanding of the precise molecular mechanisms through which Ang II contributes to oxidative stress and lens damage. While the interaction between Ang II and ROS is established, the detailed pathways and the potential variations in response among different populations remain inadequately explored. Additionally, while therapeutic interventions targeting Ang II signaling hold promise, further research is needed to validate the efficacy and safety of these approaches in clinical settings.

The significance of this research extends beyond its immediate findings, contributing to the broader field of ocular health and radiation safety. By establishing a clearer connection between Ang II, oxidative stress, and cataract formation, this study lays the groundwork for innovative therapeutic strategies aimed at mitigating the adverse effects of radiation exposure. Such advancements could significantly improve patient outcomes, enhance quality of life, and inform public health policies regarding radiation safety and prevention measures for at-risk populations.

## 9. Conclusions

As the prevalence of cataracts continues to rise, particularly among populations exposed to radiation, understanding the underlying mechanisms is crucial for developing effective preventive and therapeutic strategies. The intersection of oxidative stress and Ang II signaling presents a unique opportunity for intervention, aiming to preserve lens integrity and enhance ocular health in at-risk individuals.

Investigating the mechanisms of RICs highlights the critical roles of Ang II and ROS in cataractogenesis. Their interaction creates a compelling target for therapeutic intervention, supporting a two-pronged approach to manage and potentially reverse the effects of RICs. By addressing both pathways, future research can pave the way for effective prevention and treatment strategies that improve ocular health and quality of life for individuals exposed to ionizing radiation. Continued exploration of these avenues is essential to enhance our understanding and ability to combat this significant public health concern.
